# TEADs serve as potential prognostic biomarkers and targets for human gastric cancer

**DOI:** 10.1186/s12876-022-02386-8

**Published:** 2022-06-24

**Authors:** Meng-huan Wang, Bing-zhi Li, Yue Chen, Jie Wang

**Affiliations:** 1 Department of Fundamental and Community Nursing, School of Nursing, Nanjing Medical University, Nanjing, 211166 China; 2 Department of Nutrition and Food Safety, School of Public Health, Nanjing Medical University, Nanjing, 211166 China; 3 School of Food Science and Pharmaceutical Engineering, Nanjing Normal University, Nanjing, 210023 China

**Keywords:** TEADs, Gastric cancer, Biomarkers, Hippo pathway, Prognosis

## Abstract

TEADs are critical transcription factors that participate in the Hippo pathway. Evidence indicates the promotion role of TEADs in cancer progression. However, the role of TEADs and the expression patterns in gastric cancer remains unclear. In this study, we evaluated the expression levels of TEADs in gastric cancer samples, and the clinical outcomes of patients with high TEADs expression were observed. Co-expression and interaction analysis as well as functional enrichment analysis were further conducted to determine the potential role of TEADs in gastric cancer. These results suggested TEADs may serve as the prognostic biomarkers or therapeutic targets for gastric cancer. However, more studies are warranted to verify our findings and promote the application in gastric cancer patients.

## Introduction

Gastric cancer (GC) is the fifth leading cause of cancer deaths in the world, accounting for approximately 783,000 deaths every year [1]. Although the approaches of diagnosis and treatment for GC have been improved, the prognosis of GC patients remains poor. Studies have found that the expression of specific genes in gastric cancer tissues is different from those in adjacent normal tissues [2], and epigenetic and genetic alterations were reported in GC patients [3]. However, the molecular pathogenic mechanism of GC remains unclear. Current biomarkers are mainly used in GC screening and diagnosis, and their functions as prognostic markers of GC remain to be explored [4]. Therefore, identifying the prognostic markers of GC may provide new insights into understanding the progression of GC and contribute to the development of effective strategies to improve outcomes of the patients.

It has been reported that Hippo signaling pathway involves in the initiation and development of tumors [5]. The yes-associated protein (YAP) is a critical downstream target regulated by the Hippo signaling pathway. Emerging evidence suggests that Hippo-YAP signaling plays a key role in various tumors, including cervical cancer [6], colon cancer [7], breast cancer [8], and GC [9]. The expression of four transcriptional enhanced associate domain transcription factors (TEAD 1–4) has been observed in mammals and considered to participate in Hippo-YAP signaling. When the Hippo signaling pathway is activated, the YAP translocates into the nucleus and interacts with transcription factors, such as TEADs, thus regulating the expression of its target genes [10]. All the four TEADs proteins adopt a globular structure, consisting of a central β-sandwich fold and four α-helices on one side[11]. Studies have suggested that TEADs may be involved in epithelial-mesenchymal transition and tumor metastasis [12–14]. However, the role of TEADs in GC has yet to be fully elucidated.

Previous studies have shown evidence between the Hippo-YAP signaling and TEADs in GC [15–17]. Nevertheless, the expression of TEADs in the development and progression of GC remains unclear. In this study, we aim to evaluate the effects of TEADs expression and predict the underlying mechanisms through in-depth analysis of the expression and mutational activation of TEADs as well as their correlation with prognosis in GC patients.

## Materials and methods

### ONCOMINE analysis

ONCOMINE platform was applied to evaluate the transcriptional levels of TEADs in cancers (www.oncomine.org). The mRNA levels of TEAD4 were compared between clinical cancer specimens and the normal controls with Student's t-test. The significant difference was taken as p < 0.05.

### GEPIA database analysis

GEPIA database is a web server for analyzing the RNA expression data based on 9,736 tumors and 8587 normal samples from TCGA and GTEx projects (http://gepia.cancer-pku.cn/index.html). Here, the GEPIA database was used to compare the expression levels of TEADs in stomach adenocarcinoma and normal samples.

### The Kaplan–Meier plotter analysis

The survival analysis was conducted with Kaplan–Meier plotter (www.kmplot.com), an online database including information on gene expression and related survival of patients with GC. Sources for the databases include GEO, EGA, and TCGA. In this study, the mRNA and survival data of GC patients in this database were used. After being divided into high expression and low expression groups based on the expression levels of TEADs, the overall survival (OS), progression-free survival (FP), and post-progression survival (PPS) of GC patients were observed.

### TCGA data and cBioPortal analysis

TCGA projects provide sequencing and pathological data of human cancers, and cBioPortal is a tool developed for data analysis of TCGA. Dataset of stomach adenocarcinoma (TCGA, Firehose Legacy) containing 478 samples was included for genetic alterations analysis, including mutation, amplification, deep deletion, mRNA high, mRNA low, and multiple alterations. Co-expression analyses were conducted with spearman’s correlation based on the mRNA expression data of GC patients.

### STRING database analysis

Protein–protein interaction analysis was conducted with the STRING database (https://cn.string-db.org/). Protein interactions of TEADs in homo sapiens were predicted, and the network of proteins was generated.

### Functional Enrichment Analysis

Functions of TEADs and genes significantly associated with TEAD 1–4 expression (r > 0.5) were analyzed by Gene Ontology (GO) and Kyoto Encyclopedia of Genes and Genomes (KEGG) with Metascape, an online tool for gene annotation. Biological processes, cellular components, and molecular functions were included in GO analysis. KEGG analysis was conducted in terms at least with 3 overlaps, p < 0.05, and enrichment factor > 1.5, and the top 20 clusters with their representative enriched terms were chosen based on statistical significance.

## Results

### Transcriptional levels of TEADs in GC patients

**Fig. 1:**
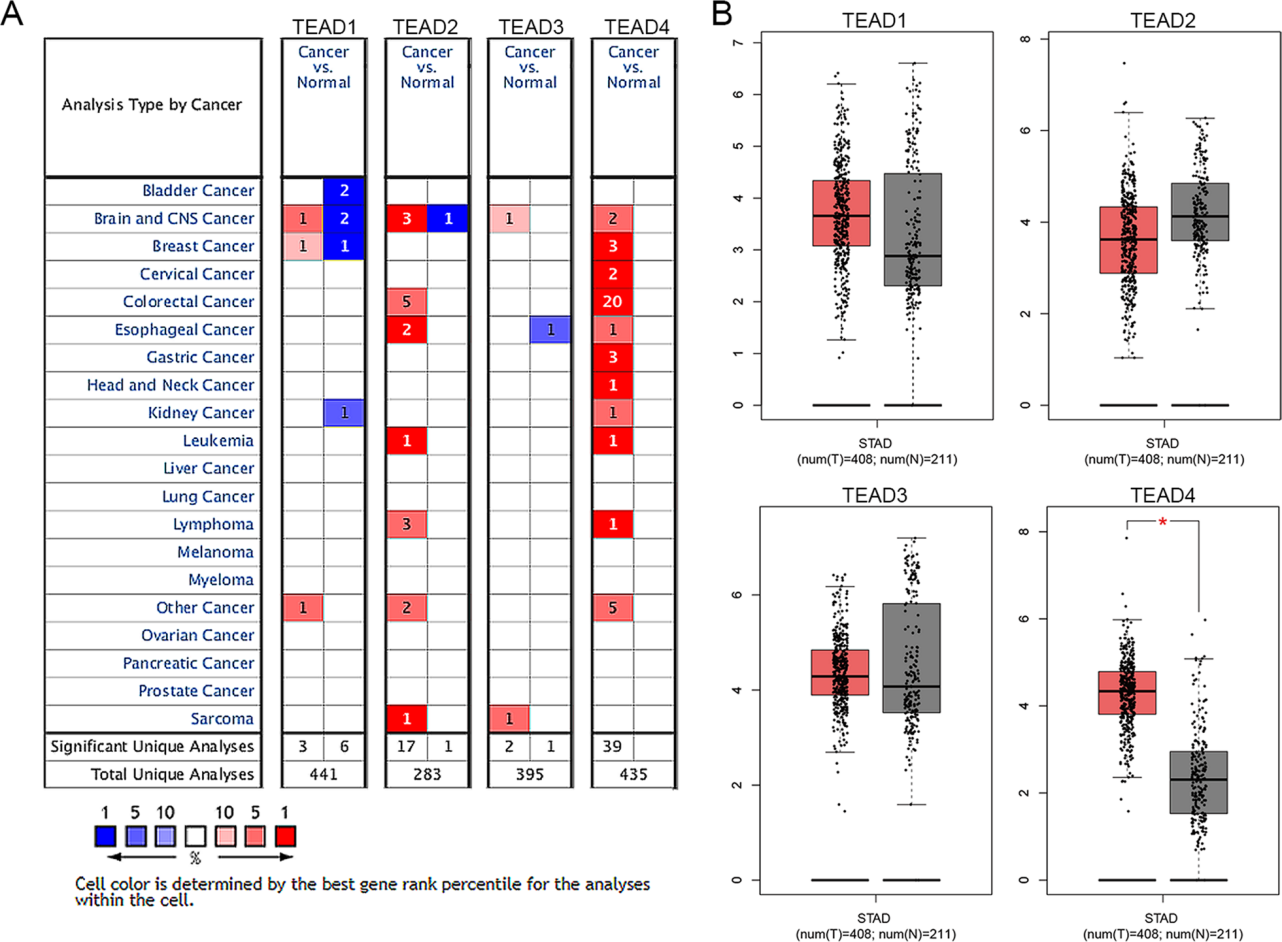
Transcriptional levels of TEADs in GC patients. **A** Transcriptional levels of TEADs in cancers were observed through the ONCOMINE database. **B** The expression levels of TEADs in stomach adenocarcinoma compared with normal samples were observed by using the GEPIA dataset

Considering four kinds of proteins containing the TEA domain were reported in the TEADs family, transcriptional levels of TEADs in cancers were observed through the ONCOMINE database. As shown in Fig. 1A and Table 1, the mRNA levels of TEAD4 were upregulated in patients with GC. In DErrico’s and Cho’s dataset, higher expression levels of TEAD4 were found in gastric intestinal type adenocarcinoma (fold change = 6.960) and gastric adenocarcinoma (fold change = 2.015) [18, 19]. Additionally, Wang et al. reported that TEAD4 was overexpressed in GC with a fold change of 5.185 [20]. By using the GEPIA dataset, we further compared the expression of TEADs in stomach adenocarcinoma with normal samples, and consistent with Cho’s results, the expression level of TEAD4 was about 2 folds in tumor than normal samples (Fig. 1B).

**Table 1:** The significant changes of TEADs mRNA expression between GC and stomach tissues

	Type of GC versus stomach	Fold change	P value	*t-*test	Ref
TEAD1	NA	NA	NA	NA	NA
TEAD2	NA	NA	NA	NA	NA
TEAD3	NA	NA	NA	NA	NA
TEAD4	Gastric Intestinal Type adenocarcinoma versus normal	6.960	3.23E-19	13.989	DErrico
	Gastric adenocarcinoma versus normal	2.015	5.42E-7	6.989	Cho
	Gastric cancer versus normal	5.185	1.40E-5	5.157	Wang

*Ref* reference

### The prognostic values of TEADs in GC patients

**Fig. 2:**
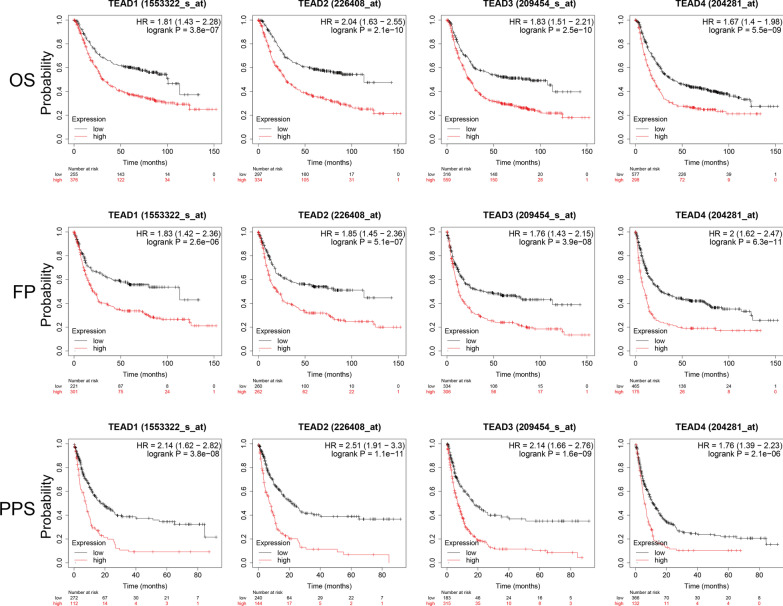
The Prognostic values of TEADs in GC patients. The Prognostic values of TEADs, including the overall survival (OS), progression-free survival (FP), and post-progression survival (PPS) in GC patients were evaluated through Kaplan–Meier plotter

The preceding data prompted us to assess the efficiency of TEADs in the survival of patients with GC, and the Kaplan–Meier plotter was used to conduct the survival analysis. As shown in Fig. 2, the overall survival (OS), progression-free survival (FP), and post-progression survival (PPS) were observed in patients with GC. Surprisingly, we found that the increased mRNA levels of TEAD 1, 2, 3, and 4 were significantly associated with OS, FP, and PPS in GC patients (p < 0.05), and patients with high mRNA levels of TEAD 1, 2, 3 and 4 were predicted to have lower OS, FP and PPS (Fig. 2).

### Co-expression and interaction of TEADs in GC patients

**Fig. 3:**
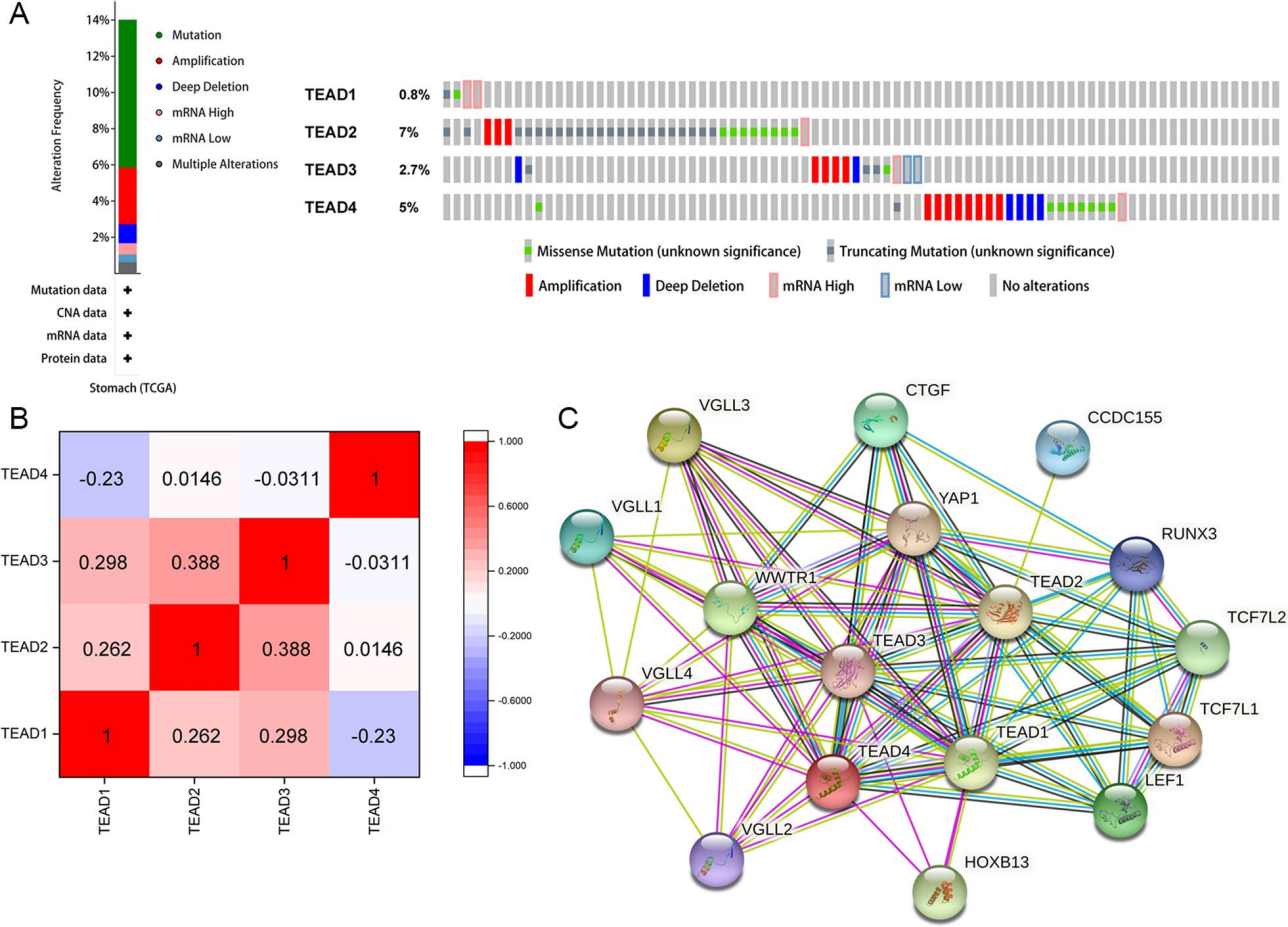
Co-expression and interaction of TEADs in GC patients. **A** The genetic alterations of TEADs in patients with GC. **B** The correlations between mRNA levels of TEADs. **C** The protein–protein interactions of TEADs were predicted with the STRING database

To evaluate the genetic alterations of TEADs in patients with GC, the cBioPortal online tool was used to analyze the TCGA dataset. TEADs were altered about 14% (67 samples out of 478 samples) in patients with stomach adenocarcinoma, and the proportion of amplification in genetic alterations was 30% for TEAD3 and 36% for TEAD4 (Fig. 3A). The correlations between mRNA levels of TEADs were also conducted through the cBioPortal online tool, and spearman’s correlation was included. As shown in Fig. 3B, a significantly positive correlation was only observed between TAED2 and TEAD3. Meanwhile, the protein–protein interactions of TEADs were predicted by using the STRING database (Fig. 3C).

### Functional enrichment analysis of TEADs network in GC patients

**Fig. 4:**
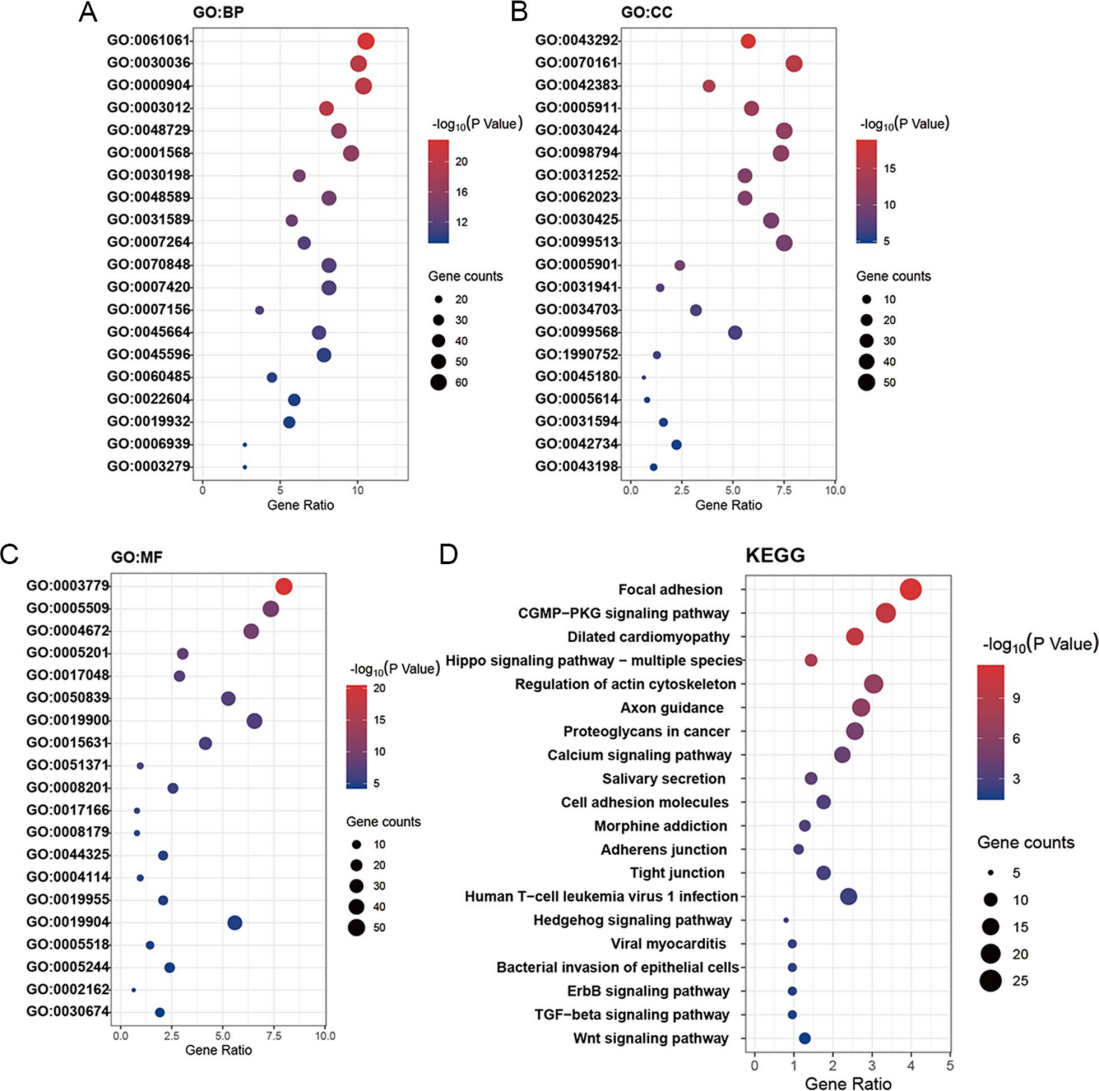
Functional enrichment analysis of TEADs network in GC patients. The functions of TEADs and genes significantly associated with TEADs alterations were predicted including **A** biological processes, **B** cellular components, **C** molecular functions. **D** KEGG analysis of TEADs and genes significantly associated with TEADs alterations

The significantly positive related genes of TEADs were then included for functional enrichment analysis. The functions of these genes were predicted by gene ontology (GO) enrichment analysis based on their biological processes, cellular components, and molecular functions (Fig. 4A–C). According to the GO biological processes analysis, GO: 0000904 (cell morphogenesis involved in differentiation), GO: 0048729 (tissue morphogenesis), GO: 0048589 (developmental growth), and GO: 0045596 (negative regulation of cell differentiation) were significantly associated with TEADs alteration in GC. For molecular functions, results were mainly related to protein binding, including GO: 0050839 (cell adhesion molecule binding), GO: 0019904 (protein domain specific binding) and GO: 0030674 (protein binding, bridging).

### Predicted pathways related to TEADs in GC patients

Kyoto Encyclopedia of Genes and Genomes (KEGG) analysis was then applied to define the pathways related to TEADs in GC patients. As shown in Fig. 4D, well-known pathways enriched in cancers, especially pathways involved in intercellular junction and cancer stem cells, were observed in the top 20 pathways significantly associated with TEADs alternations. Among these pathways, the Hippo signaling pathway, Hedgehog signaling pathway, TGF-beta signaling pathway, and Wnt signaling pathway were established as cancer stem cell pathways in recent years.

## Discussion

It was reported that TEADs are expressed in various malignancies, and may play a crucial role in tumor progression [17, 21]. Subsequent studies showed the association between TEADs expression and proliferation, regeneration as well as metastasis of cancer [22, 23]. However, the exact roles of the TEADs family in GC are still unknown. In this study, we attempted to systemically analyze the expression patterns, genetic alterations, prognostic values, and potential functions of TEADs for GC patients.

TEAD1, the most studied member of TEADs, is known to promote cell proliferation and migration in tumor progression [13, 24, 25]. The overexpression of TEAD1 was found to contribute to cell invasion and migration in endometrial cancer [26]. Besides, it was shown that TEAD1 is critical for maintaining over-activated YAP-TEAD1 signal in GC, which can promote the development of GC [27]. Here, we found that high mRNA levels of TEAD1 were observed in 0.4% of GC patients, and consistent with the previous studies, overexpression of TEAD1 positively correlated to poor prognosis in OS, FP, and PPS.

TEAD2 is a key regulator of epithelial-mesenchymal transition and tumorigenesis [28, 29]. Previous studies showed that the expression of TEAD2 was enriched in the nucleus and directed a predominant nuclear localization of YAP via the formation of TEAD2-YAP complex in the process of epithelial-mesenchymal transition of breast cancer cells [28]. Besides, the expression of TEAD2 is associated with a poor prognosis in hepatocellular carcinoma patients, which can be considered as a therapeutic target of hepatocellular carcinoma [30]. However, there are little data about the effects of TEAD2 in GC. In this study, genetic alterations including amplification, mRNA high, and mutation of TEAD2 were observed in 8% of GC patients. Although the proportion was not high, the outcomes of patients with high TEAD2 levels exhibited reduced OS, FP, and PPS.

TEAD3 is another member of the TEADs family with the least exploration. A recent study indicated that YAP-TEAD3 signaling was closely correlated with cardiomyocyte differentiation of human-induced pluripotent stem cells [31]. More recently, TEAD3 was reported to involve in metastasis and associated with a poor prognosis in pancreatic ductal adenocarcinoma [32]. In GC, evidence about the association between TEAD3 overexpression and poor prognosis in GC patients was provided in the present study.

TEAD4 regulates many biological processes including cell proliferation, tissue regeneration, and stem cell maintenance in various cell types [33]. It was reported that glucocorticoid-activated TEAD4 promoted maintenance, metastasis, and chemoresistance of breast cancer stem cells, high expression of TEAD4 predicted a poor outcome in breast cancer patients [34]. Moreover, a reduced level of TEAD4 promoter methylation in GC was significantly associated with poor outcomes, including larger tumor size and lower survival rates [35]. Through comparing the expression levels of TEAD4 in cancer versus normal tissue in both the ONCOMINE database and GEPIA dataset, we suggested that TEAD4 may participate in tumorigenesis in GC. Further survival analysis showed the poor prognosis of patients with high TEAD4 levels. Amplification was also observed in genomes of GC patients.

Studies have shown that the four members of the TEAD family are jointly involved in tumor proliferation, generation, and metastasis [17, 23, 36]. For example, Hippo pathway genes YAP1, TEAD1, and TEAD4 may jointly modulate the survival of cutaneous melanoma patients [37]. Besides, it was shown that TEAD1 and TEAD3 play redundant roles in the regulation of human epidermal proliferation [38]. Here, the cross-talk of TEADs was evaluated in both mRNA correlations and protein–protein interactions. Although we have recognized that TEADs are closely associated with the development of GC, the cross-talk of TEADs remained unclear. Based on the present results, further studies may focus on the regulatory effects of TEADs with each other.

In this study, expression levels and clinical outcomes of TEADs in GC patients were evaluated. The percentage of TEADs genetic alterations was calculated, and co-expression analyses were performed. We also constructed a network of TEADs with their neighboring genes. The functional analysis suggested that TEADs may play an important role in the development of tumors, and cancer stem cell-related pathways caught our attention. Our results indicated that TEADs may serve as the prognostic indicators or therapeutic targets in GC. Although the prognostic roles of TEADs were established, more studies are warranted to evaluate the regulatory effects of TEADs and their mechanism in GC.

## Data Availability

All the data analyzed in this study are derived from online database as listed in the Materials and methods part. Details are available from the corresponding author on reasonable request.
